# Increasing the Size of the Microbial Biomass Altered Bacterial Community Structure which Enhances Plant Phosphorus Uptake

**DOI:** 10.1371/journal.pone.0166062

**Published:** 2016-11-28

**Authors:** Pu Shen, Daniel Vaughan Murphy, Suman J. George, Hazel Lapis-Gaza, Minggang Xu, Deirdre Bridget Gleeson

**Affiliations:** 1 Soil Biology and Molecular Ecology Group, School of Earth and Environment, Faculty of Science, The University of Western Australia, Crawley, Perth, Western Australia, Australia; 2 National Engineering Laboratory for Improving Quality of Arable Land, Institute of Agricultural Resources and Regional Planning, Chinese Academy of Agricultural Sciences, P. R. China; Free University of Bozen/Bolzano, ITALY

## Abstract

Agricultural production can be limited by low phosphorus (P) availability, with soil P being constrained by sorption and precipitation reactions making it less available for plant uptake. There are strong links between carbon (C) and nitrogen (N) availability and P cycling within soil P pools, with microorganisms being an integral component of soil P cycling mediating the availability of P to plants. Here we tested a conceptual model that proposes (i) the addition of readily-available organic substrates would increase the size of the microbial biomass thus exhausting the pool of easily-available P and (ii) this would cause the microbial biomass to access P from more recalcitrant pools. In this model it is hypothesised that the size of the microbial population is regulating access to less available P rather than the diversity of organisms contained within this biomass. To test this hypothesis we added mixtures of simple organic compounds that reflect typical root exudates at different C:N ratios to a soil microcosm experiment and assessed changes in soil P pools, microbial biomass and bacterial diversity measures. We report that low C:N ratio (C:N = 12.5:1) artificial root exudates increased the size of the microbial biomass while high C:N ratio (C:N = 50:1) artificial root exudates did not result in a similar increase in microbial biomass. Interestingly, addition of the root exudates did not alter bacterial diversity (measured via univariate diversity indices) but did alter bacterial community structure. Where C, N and P supply was sufficient to support plant growth the increase observed in microbial biomass occurred with a concurrent increase in plant yield.

## Introduction

Phosphorus (P) is essential for plant growth and even though soils contain a large amount of total P, only a small fraction is available for plant uptake [1]. P fertilisers, which are mainly obtained from rock phosphate, are used in commercial agriculture to overcome P deficiencies in soil with farmers often applying P fertilisers that exceed plant requirements [2]. In the year of application only 10–30% of fertiliser P applied is taken up by the plant as most of the P applied rapidly fixes to soil clay minerals or alternatively precipitates into less available P forms [3]. Plants generally obtain P as orthophosphate (predominately as HPO_4_^2–^ and H_2_PO_4_^–^) through the use of P transporters contained within plant cell roots [1]. In most soils the soil solution concentration of orthophosphate is low and must be continually replaced with P from other soil P pools to ensure there is no P constraint to plant growth [4]. Within soil, P exists as (1) inorganic P (P_i_) which can be adsorbed to soil mineral surfaces or precipitated P which is largely unavailable or (2) organic P (P_o_) which is associated with organic matter or contained with the microbial biomass. These soil P pools are broadly grouped as readily, moderately and sparingly (recalcitrant) available pools [5]. P can be extensively sorbed or precipitated in soil and over time this increases the recalcitrant pools of soil P [6, 7]. These recalcitrant pools offer significant potential to increase plant available P if it were possible to release P from these pools into more available P pools [7].

It has been suggested that plants mediate the availability of P through release of organic anions (e.g. oxalate or citrate) that assist in mobilising both organic and inorganic P [8, 9]. A number of studies support this view for example Pearse et al. [8] reported an increase in organic anion release from P-deficient plants and where plants were engineered to release more organic anions they were able to access P from more recalcitrant pools [9]. However, the direct evidence supporting the role of organic anions is not clear. Ryan and colleagues [10] attempted to address this by assessing whether citrate efflux from roots could improve P uptake using near-isogenic lines of wheat (one cultivar which secretes citrate constitutively and one which does not). They concluded that the specific ability to exude citrate resulted in little competitive advantage and that it was likely that the citrate-effluxing cultivar had other advantages that contributed to its higher yield in P-deficient soils.

A second option to increase cycling of P from more recalcitrant pools into more available P pools is to consider how microbial cycling of recalcitrant P (both P_i_ and P_o_) can be manipulated to increase plant available P. Microorganisms play a role in mediating the availability of nutrients to plants via two main avenues; (1) an increase in the size of the microbial biomass can result in the accumulation of a significant pool of P (2) the microbial biomass can drive the transformation of P from recalcitrant to more available P pools for plant uptake [11, 12, 13, 14]. In general the soil microbial biomass is better able to obtain P from more recalcitrant pools and competes more effectively than plants for more available P pools [15, 16, 17]. Microbial P can account for between 2 and 10% of total soil P [18] and is readily impacted by environmental factors for example soil carbon (C) and nitrogen (N) availability. Research has shown that microbial P increases in response to additions of C and N; this increase in microbial P occurs concurrently with a decrease in soil orthophosphate concentration—this occurs in soils regardless of whether the soil is P limited or not [19, 20]. Microorganisms can potentially out-compete plants for available P and may immobilise a substantial proportion of soil P that will be unavailable to plants in the short term. In time, as the microbial biomass turns over, this microbial pool of P will become available to plants; thus the P immobilised in the microbial biomass could represent both an important potentially available pool of P as well as a mechanism for regulating P supply [21, 22, 11]. The mean annual flux of P through the microbial biomass has been reported to be in the region of 23 kg P ha^–1^ yr^–1^ for grassland soils and 7 kg P ha^–1^ yr^–1^ for arable soils [23] and is considered to be faster than the turnover time of C. Thus the microbial biomass can be considered a potentially significant contributor to plant P nutrition. Olander and Vitousek [22] showed that immobilisation of tracer P was greater where microbial activity was also at its peak thus indicating that the microbial pool may exert significant control over soil P cycling.

Early work by McGill and Cole [24] and Smeck [6] proposed that biological P cycling occurred independently of C and N availability. However, we now know that there are strong links between C and N availability and P cycling [25]. Here we focus on the drivers of P mobilisation and address a conceptual model, presented by Guppy and McLaughlin [7], that suggests that addition of organic material that is easily decomposed (e.g. plant residue with a low lignin content and low ratio of carbon-to-nitrogen (C:N) ratio) [26,27]) will cause a significant increase in microbial biomass. This microbial biomass increase will likely favour fast growing organisms, particularly bacteria, and potentially result in lower microbial diversity. It has been proposed that this increase in microbial biomass will consume the immediately available P pools and that consequently there may be microbially driven release of P from more recalcitrant pools to meet the needs of the growing biomass. Thus recalcitrant P maybe taken-up by the microbial biomass and form part of an enhanced P cycle. Increases in microbial biomass can be achieved by supplying either organic matter that is easily decomposed or by supplying C and N in easily-available form as root exudates.

The role of root exudates in influencing the structure and function of soil bacterial communities is widely recognised [28, 29, 30] but their potential role in influencing P cycling via the microbial biomass pool is not clear. Easily available C and N likely leads to shifts in microbial community composition toward an increased abundance of fast growing organisms that prefer nutrient rich environments (e.g. Bacteriodetes and Proteobacteria) which may have implications for P cycling, particularly if P solubilisers are enhanced for example the family Pseudomonadaceae (phylum Protobacteria). In addition to impacts of exudate C:N ratios the form of P will also have an effect on bacterial communities, in particular rock P added to soil has previously been shown to alter the bacterial community structure and this change was related to physicochemical properties of rock P [31,32]. Thus the combined effect of C and N availability and P source on microbial biomass and associated communities will likely have an influence on soil P availability to plants.

Here we tested the hypothesis that organic inputs with a low C:N ratio would increase the microbial biomass, but not its diversity, until it exhausted the supply of easily-available P forcing it to access P from more recalcitrant soil pools. In particular this hypothesis suggests that the size of the biomass is driving access to less available P rather than the diversity of organisms contained within this biomass. To test the hypothesis, we added mixtures of simple organic compounds representative at different C:N ratios to a soil microcosm experiment We designed our simple organic compounds to reflect those commonly found in root exudates [28, 30] and amended with a low C:N ratio organic substrate (C:N = 12:1) and a high C:N ratio organic substrate (C:N = 50:1) to compare contrasting effects of low and high C:N ratio root exudate substrates on soil microbial communities associated with P cycling in terms of their biomass, univariate diversity measures and community structure.

## Materials and Methods

### Soil

Soil (0–10 cm) was collected from the uncultivated Ap horizon of a wheat paddock near the town of Beverley in the wheatbelt of Western Australia (32° 2' 44.19" S, 117° 9' 54.26" E). The study was carried out on private land–the owner of this property gave permission to conduct the study on this site. The field study did not involve endangered or protected species. The soil is described as sand (94.9% sand, 3.7% clay and 1.4% silt) with a pH (CaCl_2_) of 5.0. Total soil C and N were 0.47 and 0.04% respectively resulting in a C:N ratio of 12.4. Total soil P was 42.0 mg kg^-1^ dry soil with a plant available P of 5.2 mg Colwell P kg^-1^ dry soil.

### Microcosm design

Microcosms were prepared by placing 1.5 kg soil (sieved to <4 mm) into pots with a 12.5 cm diameter and 10cm height. Three sets of P conditions were imposed: (1) No P treatment (NP) where soil was not amended with P during the course of the experiment (i.e. control), (2) Rock P treatment (RP) where RP (P 16.5%; Ca 38%; S 0.4%; K 0.15% and Mg 0.15%;) was added at a rate 130 g kg^-1^ soil (i.e. 13% w/w) prior to the experiment commencing resulting in a total of 30.75 g P applied as rock P per pot, and (3) Solution P treatment (SP) where 20.5 mg K_2_HPO_4_-P kg^-1^ soil was added every four weeks resulting in a total of 153.75 mg P being applied per pot. P application rates were different to reflect the difference in available P between easily available solution P and the less available P contained within rock P. Basal nutrients (with the exception of P) were added to all pots at the start of the experiment at concentrations (mg kg^−1^ soil) similar to that of Damon and Rengel [33]: N (33), K (88), Mg (4), Mn (3), Zn (2), Mo (0.1), Cu (0.5), B (0.1). Ryegrass (*Lolium rigidum* cv. *Tetila*) was germinated and transplanted after two weeks into each of the above sets of experimental pots at a rate of five seedlings per pot. A full set of pots was established for each of two specific root exudate treatments that were to be imposed over the duration of the experiment.

Experimental pots were amended with artificial root exudate organic compounds during the course of the 20-week experiment. We imposed these treatments in a planted system to ensure that the microbial community was continually exposed to our regime of desired C:N ratio rather than rely on root exudates from the planted ryegrass. The rate of amendment was a total of 1000 mg C kg^–1^ soil for each 4-week period and added as follows: 100, 200, 300 and 400 mg C kg^–1^ soil for weeks 1, 2, 3 and 4 respectively. This application regime was repeated every 4 weeks throughout the experiment and was chosen to mirror plant requirements as plant biomass was harvested at the end of each 4 week cycle. Thus the root exudate amendment was increased week-by-week in each 4-week period to account for increasing plant requirements. Our design aimed to compare the effect of low C:N ratio (12.5:1) root exudates with high C:N ratio (50:1) root exudates on soil characteristics (mineral N, Colwell P, soil P fractions), plant characteristics (biomass, plant P content) and microbial characteristics (MB-C, MB-P, 16S rRNA gene abundance) as well as microbial diversity and community structure. The experiment was not designed to test whether amendment with artificial root exudates would have an effect as this has already been established (e.g. [28]) and thus our treatments only included application of different C:N ratios and did not include a non-amended treatment. Artificial root exudate organic compounds with a C:N ratio of 12.5:1 and 50:1 were prepared for use in the experiment. Artificial root exudate solutions, described as constituents of root exudates [34, 28], were prepared in distilled water. The organic amendment solution with a C:N ratio of 12.5:1 contained 29.78 mM glucose, 29.78 mM fructose, 14.89 mM sucrose, 5.49 mM citric acid, 10.98 mM lactic acid, 8.24 mM succinic acid, 21.97 mM alanine, 21.97 mM serine and 13.18 mM glutamic acid. The organic amendment solution with a C:N ratio of 50:1 contained 29.78 mM glucose, 29.78 mM fructose, 14.89 mM sucrose, 13.73 mM citric acid, 27.46 mM lactic acid, 20.60 mM succinic acid, 5.49 mM alanine, 5.49 mM serine and 3.30 mM glutamic acid. Solutions were designed to maintain the same C level with the amount of N available differing between C:N ratio treatments. Artificial root exudate solutions (hereafter referred to as exudates) were applied to pots at the time of watering by pipetting a specified volume (7.5 ml) evenly onto the top of each pot which was then followed by water required to maintain pots at 50% of water holding capacity.

The experimental design consisted of three P treatments (NP, RP and SP as described above), two C:N ratio treatments (C:N = 12.5:1 and C:N = 50:1) applied as described above with three replicates of each (n = 3). This experimental design resulted in 18 pots being destructively harvested at each of 6 time points resulting in a total of 108 experimental units. All pots were fully randomised in a glasshouse where the temperature was 22.6/17.1°C day/night and relative humidity was maintained at 64%. Pots were regularly checked and adjusted to 50% of soil water holding capacity with distilled water over the course of 20 weeks and had weekly amendments of C:N substrate mixes giving a C:N ratio of 12.5:1 or 50:1. Experimental pots were destructively harvested every 4 weeks over the course of 20 weeks. At each destructive sampling time point plant shoot material was first removed, root material was then removed from each pot and the remaining soil was sieved (4 mm) to homogenise it prior to sub-sampling for further soil analyses.

### Soil and plant analyses

#### Soil analyses

After shoot and root material was removed the sieved soil was collected for analysis after each 4-week interval for soil Colwell P, mineral N, microbial biomass-C and -P (MB-C and MB-P); 10 g was preserved in liquid nitrogen and stored at –80°C for analysis of the abundance (quantitative polymerase chain reaction–qPCR) and diversity (ion torrent barcoded sequencing) of the bacterial community. Additional samples were collected after 20 weeks for measurement of soil P fractions.

#### Soil Colwell P and P fractions

Plant-available P was measured by 0.5 M NaHCO_3_ extractable P (Colwell P [35]; adjusted to pH 8.5 using NaOH). A soil:solution ratio of 1:100 was used for an extraction time of 16 h at 25°C on an end-over-end shaker. Samples and standards were read at 882 nm on a spectrophotometer (Shimadzu). Standards were prepared by adding different volumes of primary KH_2_PO_4_ standard solution to a matrix identical to that for the samples.

Soil samples were sequentially extracted for P using a modification of the Hedley procedure [36]. In brief, 0.5 g of finely ground (<150 μm) air-dried soil was weighed into centrifuge tubes, which were shaken for 16 h for each extractant followed by centrifugation. Based on the extractant used, forms of P (inorganic P–P_i_ and organic P–P_o_) extracted were recovered in the sequential order: resin-P_i_, NaHCO_3_-P_i_ and P_o_, NaOH-P_i_ and–P_o_, NaOH, HCl-P_i_ and residual P. These P fractions were grouped into readily-available P (resin-P_i_, and NaHCO_3_-P_i_), moderately-available P (NaHCO_3_-P_o_, NaOH-P_i_ and P_o_ and HCl-P_i_) and sparingly available P (residual P) as used by Guo and Yost [37].

#### Plant analyses

Ryegrass was separated into root and shoot components which were oven-dried at 60°C for 3 d and weighed. Sub-samples of plant material (shoot and root separately) were then finely ground (<150 μm) prior to further analysis. A 100 mg sample from each replicate was digested in a nitric and perchloric acid mixture. The molybdo-vanado-phosphate method was used to determine the P concentration in the digest [38]. Phosphorus uptake was calculated as total P content (P concentration multiplied by dried plant weight) of shoots and roots.

#### Partitioning of P pools

Partitioning of P into soil, accumulated plant biomass and microbial biomass pools using measured values was completed at the end of the 20-week incubation and expressed as percentages. Shifts in these partitions were statistically compared between exudate C:N ratios of 12.5:1 and 50:1 and run separately for each of the P treatments (NR, RP and SP).

### Microbial population and community analyses

#### Soil microbial biomass

MB-C was determined by chloroform-fumigation extraction [39]. Moist soil (10 g dry weight equivalent) was fumigated with purified chloroform for 24 h and extracted with 40 ml of 0.5 M K_2_SO_4_ for 1 h. Non-fumigated soils were extracted by the same procedure. The oxidisable-C in the soil extracts was measured by an automated TOC Analyser (Shimadzu, TOC-5000A, Japan). MB-C was calculated from the difference in total oxidisable-C between fumigated and non-fumigated soil with a correction factor of 0.45 [40].

MB-P was determined from the difference between the amount of inorganic P (P_i_) extracted by 0.5 M NaHCO_3_ (pH 8.5) from moist soil fumigated with CHCl_3_ and the amount extracted from unfumigated soil. Since some CHCI_3_-released P will be re-sorbed by soil during fumigation and extraction, an adjustment for this was made by incorporating a known quantity of P_i_ during extraction and correcting for recovery. MB-P was calculated from CHCl_3_-released P_i_ by dividing by 0.4, i.e. by assuming that 40% of the P in the biomass is rendered extractable as P_i_ by CHC1_3_ [41].

#### DNA extraction

Soil was sampled at 0, 8 and 20 weeks and duplicate DNA extractions were performed using the PowerSoil™ DNA Isolation Kit (MoBio Laboratories Inc.) following the manufacturer’s instructions. Duplicate DNA extractions for each treatment were then pooled and quantified and the DNA extract was stored at– 40°C prior to further analysis. DNA was extracted in this manner from each of three experimental replicates prior to amplicon sequencing and quantitative PCR (qPCR).

#### Quantitative PCR amplification (qPCR)

Copy numbers of 16S ribosomal genes were determined by qPCR using the Applied Biosystems 7500 platform. Each 20 μL qPCR reaction contained 10 μL of Power SYBR Green Master Mix (Applied Biosystems, Life Technologies), 0.2 μL of the forward Eub338 (5’-ACTCCTACGGGAGGCAGCAG-3’) and reverse Eub518 (5’-ATTACCGCGGCTGCTGG-3’) primers at a concentration of 10 μM, 2 μL bovine serum albumin (Ambion Ultrapure BSA, 5 mg mL^–1^), 2 μL of template DNA and 5.6 μL sterile water [42]. All PCR reactions were performed in triplicate using 8 ng template DNA for each experimental unit and were performed as described in Gleeson et al. [43]. Standard curves generated in each reaction were linear over four orders of magnitude (10^4^ to 10^7^ gene copies) with R^2^ values greater than 0.99. Efficiencies for all quantification reactions were 90–100%.

#### Amplicon sequencing and sequence analysis

For each sample, approximately 300 base pairs of the V4/5 region of the bacterial 16S rRNA gene were amplified by PCR primer set 515F (5’-GTGCCAGCMGCCGCGGTAA-3’) and 806R (5’-GGACTACHVGGGTWTCTAAT-3’) [44]. Amplicon sequencing was performed as descrbed previously [43] with no amendments. After sequencing, individual sequence reads were filtered within the PGM software to remove low quality and polyclonal sequences; sequences matching the PGM 3’ adaptor were also automatically trimmed. All PGM quality filtered data were exported as FastQ files which were split into constituent FastA and Qual files and subsequently analysed using the QIIME pipeline (www.qiime.org) as described previously [43]. Sequence data were sub-sampled to 10,000 sequences per sample to ensure comparable estimators across experimental units [45]. Raw demultiplexed reads have been deposited in the MG-RAST database under project ID 20589 (www.metagenomics.anl.gov).

### Statistical analysis

The experiment consisted of three factors: P treatment, C:N ratio of applied artificial root exudates and time, each with three replications. Univariate statistical analyses were performed using GenStat (16^th^ edition; Lawes Trust, Harpenden, UK). Analysis of variance (ANOVA) was performed to determine whether P treatment and exudate C:N ratio significantly affected a range of univariate measures including soil characteristics (mineral N, Colwell P, soil P fractions), plant characteristics (biomass, plant P content) and microbial characteristics (MB-C, MB-P, 16S rRNA gene abundance) as well as measures of univariate bacterial richness and diversity (Observed species, Shannon diversity, Chao1 and Faith’s phylogenetic diversity). Data was checked for normality and skewed data was log transformed prior to analysis. Differences between means were then assessed using Tukey's HSD test with a significance cut-off of *P* = 0.05. Bacterial 16S rRNA gene abundance were log-transformed prior to ANOVA as values spanned several orders of magnitude. To determine whether the treatments significantly impacted the bacterial community structure, multivariate statistical analyses were performed in Primer 6 (Primer-E Ltd., United Kingdom). Analyses were performed on data that had been generated using the QIIME pipeline as described above, with no transformation, and applying the Bray-Curtis measure of similarity. To visualise differences between treatments, ordinations were performed by principal coordinate (PCO) analysis. Tests of the null hypothesis, that there are no differences among *a priori*-defined groups, were performed by permutational multivariate analysis of variance (PERMANOVA). Relationships between changes in the bacterial community structure and individual soil characteristics were analysed using distance-based multivariate multiple regression (DistLM) with environmental measures subjected to a forward-selection procedure to develop a model to explain the variance in community structure data [46, 47].

## Results

### Plant biomass and plant P content

**Fig 1:**
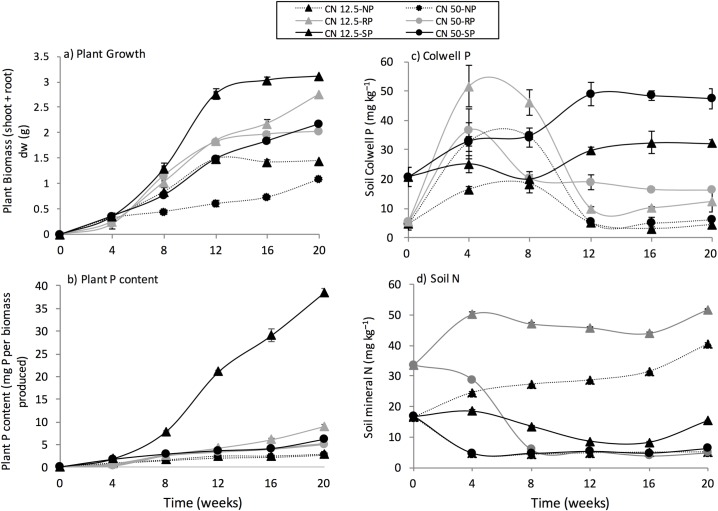
**The effect of P treatment and C:N ratio applied on (a) plant growth, (b) plant P content, (c) soil Colwell P, and (d) soil mineral N.** Dashed lines represent no P, grey lines represent rock P and solid lines represent solution P amendment—triangles represent the application of a C:N ratio of 12.5:1 and circles represent the application of a C:N ratio of 50:1. Standard error of means is shown (n = 3).

P treatment and exudate C:N ratio significantly (*P* < 0.05) affected plant growth and plant P concentration. After 20 weeks, the solution P treatment with an exudate C:N ratio of 12.5:1 produced significantly more (*P* < 0.05) plant biomass (3.11 g dry weight) than any other treatment. Within each P treatment (NP, RP, SP), application of exudates with a C:N ratio of 12.5:1 produced significantly more (*P* < 0.05) plant biomass than that when an exudate C:N ratio of 50:1 was applied (Fig 1A).

Plant P content was significantly (*P* < 0.001) affected by both P treatment and exudate C:N ratio. After 20 weeks there was a significant effect (*P* < 0.01) of exudate C:N amendment on plant P content for both RP and SP treatments but not for the NP treatment. However, the SP treatment where an exudate C:N ratio of 12.5:1 was applied had significantly higher (*P* < 0.001) plant P content (38.53 mg P per total plant biomass produced) than the average 5.20 mg P per total plant biomass produced across other treatments (Fig 1B). The rate of P uptake was not significantly different (*P* > 0.05) between C:N exudate treatments for both NP (0.035 mg P plant biomass^-1^ day^-1^ ± 0.001) and RP (0.048 mg P plant biomass^-1^ day^-1^ ± 0.005) treatments; for the SP treatment amending with a C:N ratio of 12.5:1 resulted in a significantly greater (*P* < 0.05) overall rate of P uptake at a rate of 0.178 mg P plant biomass^-1^ day^-1^) compared to 0.054 mg P plant biomass^-1^ day^-1^ where a C:N ratio of 50:1 was applied across the experimental time period.

### Soil Colwell P, P fractions and mineral N

Overall there was a significant (*P* < 0.05) effect of P treatment and exudate C:N ratio on plant-available P as measured by Colwell P (Fig 1C). Where RP was added soil Colwell P significantly increased (*P* < 0.05) initially (within the first 6 weeks) with easily released P from the added RP being released into solution and P supply being greater than plant demand for P. In time there was less P released from the RP and thus the soil Colwell P decreased significantly (*P* < 0.05) from 6 weeks until a steady state was reached about 12 weeks. In the NP treatment soil Colwell P increased significantly (*P* < 0.05) as the soil was wetted up and microbial activity commenced. Thus soil Colwell P was significantly higher (*P* < 0.05) after 6 weeks–the increase in Colwell P in the NP treatment was much less than for the RP treatment. With less P being available for release from soil organic matter and with plant growth increasing soil Colwell P decreased significantly (*P* < 0.05) from 6 weeks onwards until a steady state low soil Colwell P was reached at approximately 12 weeks. In the SP treatment where soluble P was added continuously the generally high soil Colwell P measured reflects this.

Assessing differences after the 20 week incubation there was a significant effect of exudate C:N ratio on plant-available P in the RP and SP treatments but not the NP treatment. The NP treatment had the lowest plant-available P levels at 5.3 mg Colwell P kg^-1^ dry soil while the SP treatment combined with an exudate C:N ratio of 50:1 had the highest plant-available P levels at 47.5 mg Colwell P kg^-1^ dry soil; the application of an exudate C:N ratio of 12.5:1 resulted in significantly (*P* < 0.05) less plant-available P in the SP treatment (32.2 mg Colwell P kg^-1^ dry soil). The difference in plant-available P within the RP treatments was less pronounced than the SP treatment, but still significant (*P* < 0.05) with application of an exudate C:N ratio of 50:1 resulting in 16.2 mg Colwell P kg^-1^ dry soil compared to 12.3 mg Colwell P kg^-1^ dry soil with application of an exudate C:N ratio of 12.5:1.

**Fig 2:**
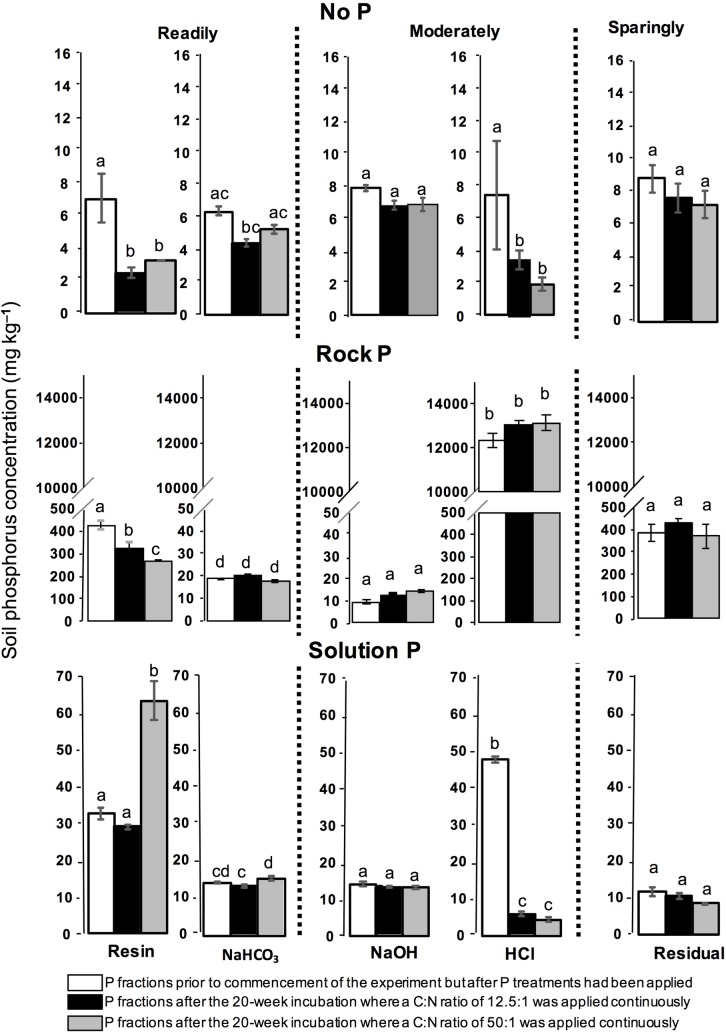
Change in soil P forms over the course of the experiment. (1) white bars represent P fractions prior to commencement of the experiment but after P treatments had been applied (2) black bars represent P fractions after the 20-week incubation where a C:N ratio of 12.5:1 was applied continuously and (3) grey bars represent P fractions after the 20-week incubation where a C:N ratio of 50:1 was applied continuously. Soil P forms were further grouped based on perceived plant P availability (readily-available P, moderately-available P and sparingly-available P). Standard error of means are shown (n = 3). Significant differences (*P* < 0.05) are indicated by different lowercase letters while those not statistically significant (*P* > 0.05) are indicated by the same lowercase letters. Different superscript letters indicate significant difference (P<0.05) in P fractions after 20 weeks following the two C:N treatments compared to initial levels as assessed by ANOVA followed by Fisher’s least significant difference (LSD).

Total P and soil P fractions were assessed at the start of the experiment and after 20 weeks of incubation with exudate C:N substrates (Fig 2). The NP treatment had the lowest soil P concentrations both at the beginning and end of the 20 weeks compared to the RP and SP treatments. For NP, there was significantly less (*P* < 0.05) resin extractable P (readily-available P) in soil at the end of the experiment (for treatments receiving exudates with C:N ratios of 12.5:1 and 50:1) compared to the start of the experiment. Resin and NaHCO_3_ extractable P fractions (readily-available P) in the NP treatment did not significantly differ (*P* > 0.05) between exudate C:N ratios of 12.5:1 and 50:1 at the end of the experiment. For RP, the HCl-extractable fraction (moderately-available P fraction) was significantly higher (*P* < 0.001) than the NaOH extractable P (moderately-available P) both at the start of the incubation and after 20 weeks of incubation with the exudate C:N ratios of 12.5:1 and 50:1. There were no significant differences (*P* > 0.01) in the NaOH or HCl-extractable fractions at the start of the experiment and after incubation with exudate C:N ratios of 12.5: or 50:1 for 20 weeks. For SP, treatments receiving exudates with a C:N ratio of 50:1 had significantly higher (*P* < 0.01) soil resin extractable P and NaHCO_3_ extractable P (*P* < 0.05) after 20 weeks when compared to soil at the start of the incubation and soil incubated for 20 weeks with an exudate C:N ratio 12.5:1.

Significantly lower levels of HCl-extractable P (*P* < 0.01) were observed for soils incubated with exudate C:N ratios 12.5:1 and 50:1 after 20 weeks compared to HCl-extractable P at the start of the incubation. The residual or sparingly available P fraction did not differ significantly (P > 0.05) between soil at the start of the incubation and soil incubated for 20 weeks with exudate C:N ratios of 12.5:1 and 50:1 for NP, RP and SP treatments (Fig 2).

After 20 weeks of incubation P treatment and exudate C:N ratio significantly affected (*P* < 0.05) soil mineral N; however the effect of P treatment was only significant where an exudate C:N ratio of 12.5:1 was applied. Within each P treatment (NP, RP, SP), soil mineral N concentration was significantly (*P* < 0.05) greater where an exudate C:N ratio of 12.5:1 was applied compared to an exudate C:N ratio of 50:1, with the greatest effect within the RP treatment (Fig 1D). Amendment with RP increased soil mineral N at the start of the microcosm from 16.74 mg N per kg dry soil (in the NP and SP treatments) to 33.60 mg N per kg dry soil (in the RP treatment only).

### Microbial biomass carbon and phosphorus and 16S rRNA gene abundance

**Fig 3:**
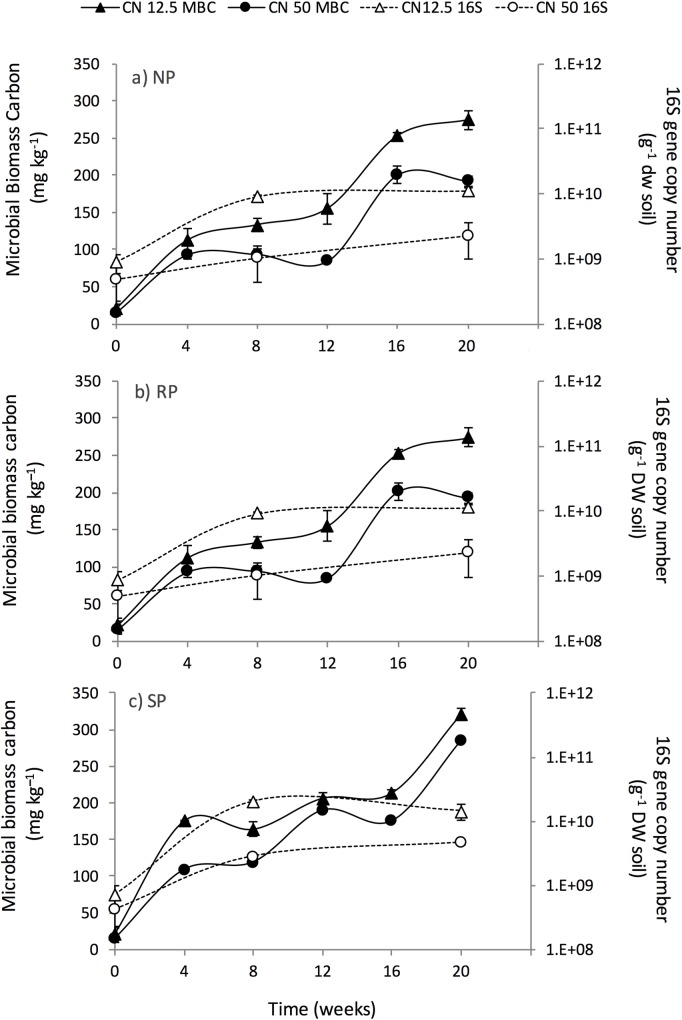
**Change in MB-C (solid lines) and 16S gene abundance (dashed lines) with (a) no P treatment (b) rock P treatment (c) solution P treatment.** Closed triangles represent MB-C where an artificial root exudate with a C:N ratio of 12.5:1 was applied; closed circles represent MB-C where an artificial root exudate C:N ratio of 50:1 was applied. Open triangles represent bacterial 16S gene abundance where an artificial root exudate C:N ratio of 12.5:1 was applied and open circles represent bacterial 16S gene abundance where an artificial root exudate C:N ratio of 50:1 was applied.

After 20 weeks P treatment and exudate C:N ratio significantly (*P* < 0.005) affected soil MB-C. The MB-C was significantly (p<0.005) higher after 20 weeks at 322 mg C kg^-1^ dry soil in the SP (with the application of a C:N ratio of 12.5:1) compared to the RP and NP treatments, with RP also having a significantly higher MB-C (274 mg C kg^-1^ dry soil weight and a C:N ratio of 12.5:1) than the NP treatment which had the lowest MB-C at 146 mg C kg^-1^ dry soil with the application of a C:N ratio of 50:1. Within each P treatment, application of an exudate C:N ratio of 12.5:1 produced significantly higher MB-C than application of a C:N ratio of 50:1 (*P* < 0.001) (Fig 3).

MB-P followed the same trend as MB-C and after 20 weeks of incubation the SP treatment (with application of an exudate C:N ratio of 12.5:1) had significantly (*P* < 0.001) higher (11 mg P kg^–1^ dry soil) MB-P than the RP (8.5 mg P kg^–1^ dry soil) and NP (6.6 mg P kg^–1^ dry soil) treatments. Within each P treatment, application of an exudate C:N ratio of 12.5:1 had significantly (*P* < 0.001) higher MB-P than application of an exudate C:N ratio of 50:1 (data not shown). Both P treatment and exudate C:N ratio also significantly (*P* < 0.001) affected bacterial 16S rRNA abundance. After 20 weeks, the SP treatment had a significantly (*P* < 0.001) greater bacterial abundance at 1.45 x 10^10^ 16S gene copies g^–1^ dry soil than the NP or RP treatments. Within each P treatment, application of an exudate C:N ratio of 12.5:1 had a significantly greater 16S rRNA abundance than application of an exudate C:N ratio of 50:1 (*P* < 0.001) (Fig 3).

### P budgets across soil, plant and microbial pools

**Fig 4:**
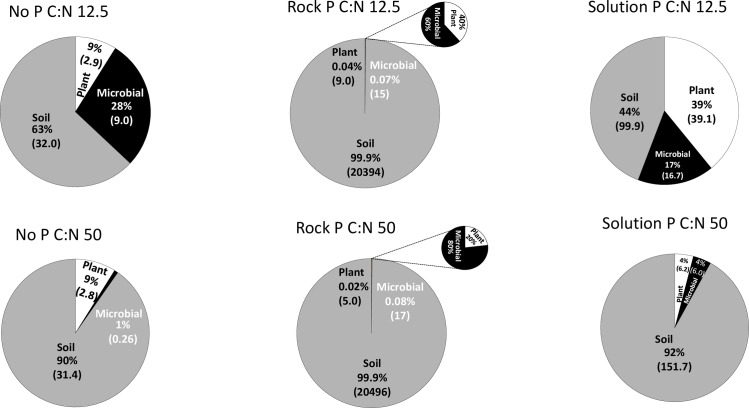
Apportionment of P in soil, accumulated plant biomass and microbial biomass pools quantified at the end of the 20-week pot trial and expressed as percentages and P content (mg P kg^–1^ soil) in parentheses.

Based on the P budgets at the end of the 20-week incubation (Fig 4), the NP treatment with application of an exudate C:N ratio of 12.5:1 had a significantly higher portion of P in the microbial P pool compared to plant biomass P (*P* < 0.05) while a significantly lower level of microbial P but similar level of plant biomass P was observed with application of an exudate C:N ratio of 50:1. In both NP and SP treatments, significantly (*P* < 0.05) more P was taken up into the plant and microbial biomass pools with application of an exudate with a C:N ratio of 12.5:1 compared to an exudate with a C:N ratio of 50:1. Unlike the NP treatment, the SP treatment with application of an exudate C:N ratio of 12.5:1 had a significantly higher portion of P in the plant biomass P compared to microbial P pool (*P* < 0.05). RP treatments for both exudate C:N ratios did not significantly differ (*P* < 0.05) between microbial and plant biomass P pools. For all NP, SP and RP treatments, soil P had significantly higher (*P* < 0.01) levels of P compared to either microbial and plant P at both exudate C:N ratios.

### Bacterial diversity and community structure

Diversity and community structure at the start of the incubation did not significantly differ (*P* > 0.1) across all measures (univariate and multivariate). As such, the results outlined in the following section detail differences observed at the end of the 20-week incubation only. The obtained sequence data represented a diverse soil community, however observed operational taxonomic unit (OTU) richness estimators did not differ significantly (*P* > 0.1) among treatments (1456 ± 281 OTUs identified) including the Chao1 richness index (3363 ± 351). Coverage estimators were generally high (Good’s coverage 0.911 ± 0.01) and not significantly different (*P* > 0.1) across treatments indicating similar deep recovery of the community constituents across the treatments. Diversity did not differ significantly (P > 0.1) across treatments as measured by the Shannon index (7.35 ± 0.37) and Faith’s Phylogenetic Diversity (107.7 ± 10.8).

**Fig 5:**
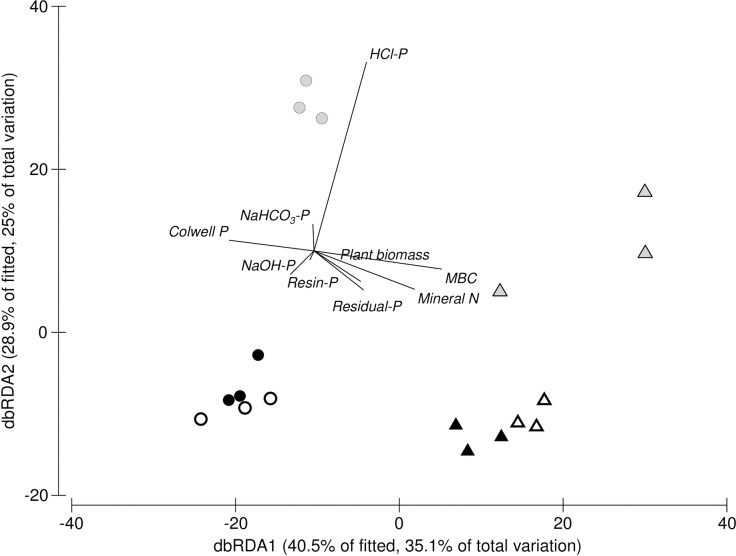
Distance-based redundancy analysis (dbRDA) of variation in bacterial community profiles as explained by environmental variables. Vectors represent correlations of variables with community structure along the first two dbRDA axes. No P amendment is represented by black symbols, rock P amendment is represented by open symbols and solution P amendment is represented by grey symbols. Application of a C:N ratio of 12.5:1 is represented by triangles and application of a C:N ratio of 50:1 is represented by circles. The values in parentheses indicate the percentages of the fitted and total variations explained by each axis.

Despite the lack of difference in univariate measures, when the data was analysed using multivariate statistics both P treatment and exudate C:N ratio had a significant (*P* < 0.01) effect on the bacterial community structure (Fig 5). Within each P treatment, the bacterial community structure where an exudate C:N ratio of 12.5:1 was applied differed significantly (*P* < 0.001) to that where an exudate C:N ratio of 50:1 was applied. For each exudate C:N ratio the community structure was significantly different (*P* < 0.01) between P treatments, however the effect was greatest for RP compared to either NP of SP where the community structure differences between exudate C:N ratios of 12.5:1 and 50:1 was not as pronounced (Fig 5). Forward-selection models identified seven soil variables which together explained 82% of the variance in the community structures data (Table 1). Mineral N alone explained 28.4% of the variation with HCl-extractable P explaining a further 23.4%, other variables combined explained the final 30.2% variation in the data set.

**Table 1:** Distance-based multivariate multiple regression showing relationships between environmental variables and bacterial community structure.

Variable (mg kg^-1^ unless otherwise listed)	*P*	Prop. (%)
Mineral N	0.001	28.4
HCl-extractable P	0.001	51.8
Plant Biomass (g)	0.008	58.8
Microbial Biomass C	0.01	67.4
Colwell P	0.03	72.6
NaHCO_3_-extractable P	0.06	76.6
Resin-P	0.1	82.0
NaOH-extractable P	NS	-
Residual P	NS	-

Data shown are from a forward-selection model where only variables that contributed significantly to the model were included. The significance of the relationship (*P*) and the cumulative percentage of variation explained (Prop.) is shown.

**Fig 6:**
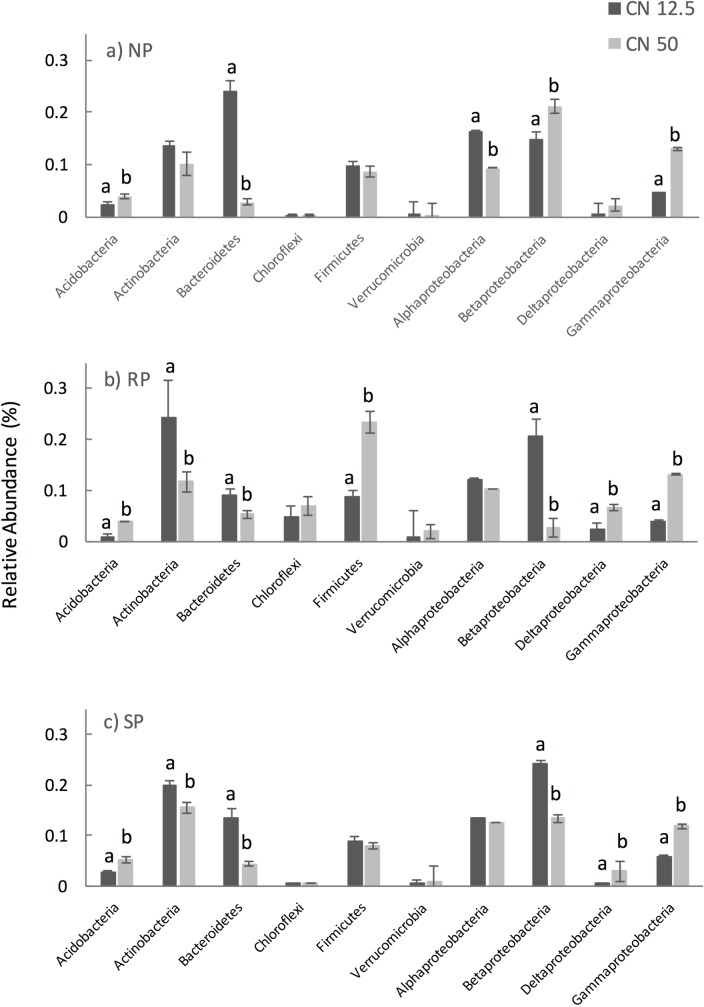
**Dominant phylum level changes between treatments at 20 weeks are shown for treatments with (a) no P treatment; (b) rock P treatment and (c) solution P treatment.** Black bars represent phylum relative abundance where a C:N ratio of 12.5:1 was applied and grey bars represent phylum relative abundance where a C:N ratio of 50:1 was applied. Standard error of means is shown (n = 3).

For all treatments after 20 weeks, Proteobacteria was the dominant phylum, followed by Actinobacteria and Bacteroidetes; in the RP treatment in particular Firmicutes were also high in relative abundance (Fig 6). Where NP was applied Bacteriodetes and Alpha-proteobacteria were significantly more abundant (*P* < 0.05) where an exudate C:N ratio of 12.5:1 was applied compared to an exudate C:N ratio of 50:1. In contrast Acidobacteria and Beta- and Gamma-proteobacteria were significantly more abundant (*P* < 0.05) where an exudate C:N ratio of 50:1 was applied compared to an exudate C:N ratio of 12.5:1. Where SP was applied again Bacteriodetes was significantly more abundant (*P* < 0.05) where an exudate C:N ratio of 12.5:1 was applied compared to an exudate C:N ratio of 50:1, however Actinobacteria, and Beta-proteobacteria were also significantly more abundant (*P* < 0.05). Only Gamma-proteobacteria were significantly more abundant (P < 0.05) where an exudate C:N ratio of 50:1 was applied compared to an exudate C:N ratio of 12.5:1. For both NP and SP there was no significant effect (*P* > 0.05) of exudate C:N ratio on Chloroflexi, Firmicutes and Verrucmicrobiota. Where RP was applied Bacteroidetes, Actinobacteria and Beta-proteobacteria were significantly more abundant (*P* < 0.05) where an exudate C:N ratio of 12.5:1 was applied and Acidobacteria Firmicutes, Delta- and Gamma-proteobacteria were significantly more abundant (*P* < 0.05) where an exudate C:N ratio of 50:1 was applied. There was no significant effect (*P* > 0.05) of exudate C:N ratio on the abundance of Alpha-proteobacteria, Chloroflexi or Verrucomicrobia (Fig 6).

## Discussion

Amendment with low C:N ratio artificial root exudates increased the size of the microbial biomass but did not impact its diversity; in contrast amendment with high C:N ratio artificial root exudates did not result in a similar increase in microbial biomass but had a similar lack of effect on diversity. This supports our hypothesis that application of a low C:N ratio artificial root exudate (i.e. 12.5:1) increased the size of the microbial biomass while also increasing plant yield, at least where P supply was sufficient to support plant growth. However, implications for P cycling were variable with no change in the size of more recalcitrant P pools under any of the experimental conditions applied over the course of our 20-week incubation time and thus our data do not support the hypothesis that measurable P was released from more recalcitrant P pools. We do report an increase in plant yield with application of a low C:N ratio artificial root exudate when combined with the supply of an easily-available P source (SP treatment) or a less readily available but significant source of P (RP treatment) which supplied the plant with its C, N and P requirements. Therefore, despite the fact that we did not observe a decrease in more recalcitrant pools *per se* we may be observing increased flux through these pools which is making more P available for plant uptake.

### Impact of artificial root exudate C:N ratio

Plant root exudates impact the abundance, structure and diversity of rhizosphere microbial communities [28, 48, 29, 30] and the use of artificial root exudates, similar to those reported herein, also impact bacterial community structure when added as a solution of varying C:N ratio [28]. A number of studies have reported that the size of the microbial biomass was impacted by the quality of resources available [48, 49] which aligns with our findings. As expected, due to amendment with easily-available C and N, we report an increase specifically in the size of the the microbial biomass (Fig 3) but no change to the bacterial diversity contained within that biomass. High C:N ratio amendments could have the potential to increase diversity within the microbial biomass [7], however this is only likely where complex amendments are used. This is likely due to greater need for organisms with more diverse biochemical pathways to break down more challenging organic amendments (e.g. wheat straw) [50]. In the current study, we added a high C:N ratio substrate in the form of an artificial root exudate which was unlikely to increase diversity in general, but could potentially impact the structure of the bacterial community when compared to the application of a lower C:N ratio of 12.5:1.

Although there was no impact of C:N ratio of the artificial root exudate substrates on univariate measures of diversity, we found that both the P treatment and C:N ratio of the artificial root exudate significantly impacted bacterial community structure. The impact of RP on bacterial community structure observed here agrees with previous findings which illustrated the impact of minerals [51, 52] and in particular rock phosphate [31, 32] on bacterial community structure. Within each P treatment there was a significant impact of C:N ratio of the artificial root exudate applied; this impact was more pronounced for RP than for the other treatments. These results demonstrate that root exudates can have a significant effect on the structure of soil bacterial communities and agrees with previous work using these root exudates [28, 29, 30]. In the current study, C input was maintained at the same level with N inputs altered (by varying carboxylic acid and amino acid concentrations). Amino acids are critically important as they play a key role in metabolic pathways; these results indicate that availability of amino acids is also critical in determining bacterial community structure as changes in amino acid concentrations in our artificial root exudates impacted bacterial community structure.

### Impact of phosphorus source

In planted systems, both plant roots and microbial biomass will initially take up the most easily-available forms of P; therefore mechanisms to increase the size of this pool are likely to result in increased plant yield. Movement of P from recalcitrant to more available P pools has been shown following the addition of easily decomposed organic material for example manures [53, 54]. Similarly, Oberson et al. [55] and Lehmann et al. [56] reported that in pasture and forest systems, respectively, the addition of high quality (low C:N ratio combined with high P content) organic matter improved P cycling in these systems. Assessing P fractions across these studies suggests that organic matter quality played a substantial role in increasing the P content of the easily available pools. However, addition of organic matter of low quality (high C:N ratio combined with low P content) did not result in movement of P from less- to more-available P pools [53, 54]. This was probably related to the decreased speed of decomposition of this poorer quality organic matter in addition to its lower P content. The increase in microbial biomass we observed on application of a low C:N ratio artificial root exudate however did not occur in conjunction with a concomitant release of sparingly available or recalcitrant P and, at the end of the 20 week incubation, there was no significant difference in the residual or sparingly available P pools regardless of P amendment (NP, RP or SP). In the more available P pools, the NaHCO_3_-extractable P pool significantly increased (P < 0.05) where SP was added compared to NP and RP treatments which is likely related to the addition of soluble P into the system as application of P fertiliser increases the labile and moderately-available P pools [57].

### Impact on specific bacterial phyla

Across all P treatments (NP, RP and SP) the relative abundances of Acidobacteria, Actinobacteria, Bacteroidetes, Beta- and Gamma-proteobacteria differed significantly between the C:N ratios of 12.5:1 and 50:1. Acidobacteria are ubiquitous in soil and include metabolically and genetically diverse organisms; it is reported that due to their metabolic and genetic versatility that they potentially play an important role in agricultural systems [58]; however information on their specific role is still lacking. Fierer and colleagues [59] reported that their increased abundance in soils with low resource availability which is consistent with our finding that they were more abundant when artificial root exudates with a C:N ratio 50:1 are applied compared to a C:N ratio of 12.5:1. Actinobacteria are not easily delineated into oligotrophic or copiotrophic organisms [59] and are characterised by the ability to mineralise soil organic matter [59, 60]. In the current study we report an increased abundance of Actinobacteria with application of a C:N ratio of 12.5:1 suggesting that the Actinobacteria present may prefer more nutrient-rich environments. Additionally, some members of the Actinobacteria are better able to solubilise rock phosphate [61, 62] which may account for their relatively high abundance in the RP treatment in the current study.

It has been reported that Beta-proteobacteria and Bacteroidetes in particular exhibit copiotrophic attributes with their relative abundances being highest in nutrient-rich environments [59, 63, 64]. In our study we also found that the relative abundance of these copiotrophic organisms was greatest where a C:N ratio of 12.5:1 was applied. In addition we observed a significant increase in the relative abundance of Delta-proteobacteria where a C:N ratio of 50:1 was applied—again these are generally considered to be oligiotrophic organisms preferring a less nutrient-rich environment [59]. For Verrucomicrobiota, a negative correlation with N content has been reported in several studies [65, 66] and members of this group are generally thought of as being oligiotrophic; in the current study we report a significant impact of C:N ratio on Verrucomicrobiota only in the RP treatment with a C:N ratio of 50:1 having a higher relative abundance of these organisms which is in line with other reports regarding the likely oligiotrophic preferences of this group. Within the RP treatment in particular Firmicutes were significantly more abundant than in the NP or SP treatments, and significantly more abundant where a C:N ratio of 50:1 was applied. This correlates with previous studies which implicate Firmicutes where rock phosphate is present [67, 68]. In the NP and RP treatments in particular we also noted an increase in the abundance of families containing known P solubilisers including Pseudomonadaceae and Bacillaceae but also those less well known for exhibiting P solubiliser activity for example Micrococcaceae and Xanthomonadaceae among others [68].

## Conclusions

Our results show that (i) amendment with artificial root exudate organic substrates of low C:N ratio increased the size of the microbial biomass and (ii) there was no P release from recalcitrant P pools (iii) where P was in sufficient supply there was a consequent increase in plant yield and plant P uptake. Where artificial root exudates with a C:N ratio of 50:1 were applied, the system quickly became N limited which decreased plant growth. We found no impact of C:N ratio on the diversity of the microbial community in terms of univariate measures; however, the overall structure of the community was impacted. We did not detect any movement of P from more recalcitrant pools into more-labile pools and although we did find P mobilised from moderately-available pools into readily available this was not related to the C:N ratio of the organic amendment. We conclude that plants had an advantage over the microbial biomass in accruing P when there was a surplus of easily-available P as opposed to the competitive advantage of microbes in maintaining larger P pools compared to plants under the limited and probably less readily-available P source of the NP treatment.
